# Effect of TTLL6 expression on CDDP sensitivity of EC109/CDDP cells in hypoxia/acidosis microenvironment

**DOI:** 10.7150/jca.47694

**Published:** 2020-09-30

**Authors:** Yang Qiu, Chao Wu, Jingyao Li, Meng Tang, Shixin Zhang, Tao Jing, Yi Liao, Haidong Wang

**Affiliations:** 1 Department of thoracic surgery, Southwest Hospital, Army Medical University (Third Military Medical University), Chongqing, China.; 2 State Key Laboratory of Silkworm Genome Biology, The Institute of Sericulture and Systems Biology, Southwest University, Chongqing, China.; 3 Department of Cardiology, Southwest Hospital, Army Medical University (Third Military Medical University), Chongqing, China.

**Keywords:** Chemotherapy, Drug resistance, EC109/CDDP, Hypoxia/Acidosis, TTLL6

## Abstract

Multidrug resistance is a major obstacle to the effective treatment of esophageal carcinoma. It occurs more readily in hypoxia and acidosis microenvironment. TTLL6 is one of Tubulin tyrosine ligase-like family members. In this study, the effect of TTLL6 on the regulation of cisplatin (CDDP) sensitivity was evaluated in CDDP-resistant esophageal carcinoma (EC) cells both *in vitro* and *in vivo*. In hypoxia/acidosis condition, overexpression of TTLL6 in EC109/CDDP cells significantly lowered the IC_50_ of CDDP and increased the CDDP-induced apoptosis; while knockdown of TTLL6 expression in EC109/CDDP cells exhibited the opposite effects. Further study showed that, mechanistically, TTLL6 was inversely correlated with ERBB2 and TOPOIIA, and positively correlated with apoptosis-associated factor Caspase 9. Furthermore, animal model confirmed that TTLL6 negatively regulated the growth of xenograft tumor after chemotherapy. Alternated expression of TTLL6 also regulated the expression of ERBB2, TOPOIIA and Caspase 9 in EC109/CDDP cells *in vivo*. In conclusion, our results suggest that TTLL6 could reverse the drug resistant of EC109/CDDP cells, it might provide a potential treatment strategy for the clinical reversing the chemotherapy resistance.

## Introduction

Esophageal cancer (EC) is one of the most common cancers in China with only about a 20% overall five-year survival rate 1-3. Although, chemotherapy is an important part of combination treatment strategies for esophageal cancer, multidrug resistance (MDR) of esophageal cancer cells to chemotherapy has been a major factor affecting the efficacy of chemotherapy and the prognosis of patients 4. One of the basic characteristics of the metabolic environment of tumor tissue is hypoxia 5. It is reported that the oxygen partial pressure (PO_2_) of normal tissues is 40 mmHg - 50 mmHg, while the tumor PO_2_ is 5 mmHg - 10 mmHg or lower 6, 7. On the other hand, the pH value of the microenvironment plays an important role in the occurrence and development of tumors, especially drug resistance. It has been reported that the extracellular pH (pHe) of tumors is from 6.5 to 7.0 (the lowest can reach 6.0) 8. It is more specific that the fermentation and decomposition of food residue produce a lot of acidic substances. Therefore, the human esophageal cancer cells and the formation of MDR are in a hypoxia/acidosis microenvironment. However, previous studies have not attached the importance to the special micro-environment in which EC cells were located. Therefore, studying the effect of hypoxia/acidosis microenvironment on EC cells will help us to understand the biological behavior of tumor more practically and improve the prognosis of patients.

Tubulin-tyrosine ligase (TTL) is critical for maintaining the function of microtubule which involves in cell movement, division, and shape maintenance. It is also reported that TTL plays important roles in cell apoptosis 9. TTL post-translationally catalyzes the addition of a tyrosine to the C-terminal end of detyrosinated alpha-tubulin, and tyrosine carboxypeptidase (TCP) can reverse this process 10. Under hypoxia and high concentration of reactive nitrogen condition, a large number of peroxynitrite (ONOO-) are produced in tissue, which can cause the nitrification of tyrosine to form 3-nitrotyrosine (3-NT). 3-NT can also be connected to microtubule with the assistance of TTL to form nitrified tyrosine microtubules. However, this reaction cannot be reversed by TCP resulting in damaging the microtubule network and eventually causing cell apoptosis 11. Tubulin-tyrosine-ligase-like-6 (TTLL6) is a member of the TTL family. The human TTLL6 gene is located in the chromosomal locus17q21.32 coding a conservation domain of TTL 12. TTLL6 is mainly expressed in the testicles and participates in apoptosis-related spermatogenesis and differentiation of reproduction organ 13, 14. In our previous study, we have found that expression of TTLL6 significantly increased in reversed cisplatin resistant EC cells (EC109/CDDP/WIG-1). As we mentioned before, human EC cells are growth in a hypoxia/acidosis and high content of active nitrogen microenvironment where the tyrosine can be modified to produce a large amount of 3-NT. Therefore, we believe, in such an environment, enhancement of TTLL6 expression in drug resistant EC cells may increase the efficacy of chemotherapy or lead to reversal of drug resistance through activating apoptosis signaling pathway.

In our study, we investigated the effect of TTLL6 on CDDP sensitivity in EC109/CDDP cells both *in vitro* and *in vivo*. We found that increasing/silencing the expression of TTLL6 enhanced/weakened the drug-induced cell apoptosis of EC109/CDDP in hypoxia/acidosis but not neutral microenvironment, and this efficacy might result from altering the expression of several apoptosis-related genes 15. We also observed a suppression effect of xenograft tumor growth in TTLL6-overexpressing EC109/CDDP cells' mouse molded and a promoting effect in TTLL6-konckdown EC109/CDDP cells' mouse molded after CDDP treatment in both hypoxia/acidosis and neutral microenvironment. Compared to the neutral microenvironment group the effect of alternated TTLL6 expression on tumor growth in hypoxia/acidosis microenvironment group were more significant. To our knowledge, this study firstly revealed the effects of TTLL6 on CDDP sensitivity in EC cells in different culture conditions. Our result demonstrates a novel understanding of the mechanism of CDDP resistance and may provide an intervention strategy for drug resistance reversal of EC chemotherapy.

## Materials and Methods

### Plasmids

Human TTLL6 coding DNA was amplified by PCR using cDNA prepared from EC/109 cells and subcloned into pLenti-CMV-GFP-Puro (Addgene) between BamH I and Sal I restriction sites to form pLenti-CMV-TTLL6 vector. To stably silence TTLL6 expression, shRNA coding DNA sequence was subcloned into plasmid pLKO.1-TRC cloning vector (Addgene) between Age I and EcoRI sites to form pLKO.1-shRNA/TTLL6 vector and scrambled non-sense shRNA sequence was used as control (pLKO.1-shRNA/control).

### Cell culture

Human esophageal carcinoma cells EC109, EC109/CDDP and HEK-293T cells were grown in DMEM medium (Corning Cellgro) containing 10% fetal bovine serum (HyClone), 100 U/ml penicillin, and 100 mg/ml streptomycin (Corning Cellgro) at 37 °C with 5% CO_2_. For stably selected EC109/CDDP-shRNA/control, EC109/CDDP-shRNA/TTLL6, EC109/CDDP-GFP and EC109/CDDP-TTLL6 cells the medium was supplemented with 2.0 µg/mL puromycin (Sigma-Aldrich). Hypoxia/acidosis microenvironment was created by decreasing the O_2_ concentration to 1% in a hypoxic chamber (MACS VA500 microaerophilic workstation, Don Whitley Scientific, Bingley, UK) with 5% CO_2_ and residual N_2_ and adjusting the culture medium to pH 4.0 with 0.1M of hydrochloric acid solution.

### EC109/CDDP cell line and IC_50_ determination

Cisplatin-resistant EC cell line EC109/CDDP cells were established by culturing EC109 cells with crescent concentration of cisplatin (form 0.1 µg/mL to 0.5 µg/mL) for 9 months, and then maintained in the absence of drug for 3 weeks. The IC_50_ was determined by seeding indicated cells on 96 well plate overnight and then treating cells with increasing concentration of drug for 72 hours; then MTT analysist was used to measure the living cell number to assay the IC_50_.

### Cell invasion analysis

The invasion ability of EC109/CDDP cells was analyzed by transwell assay. Briefly, the transwell chambers (Corning, USA) were precoated with Matrigel (BD, USA). Next, 5×10^4^ indicated cells were seeded in the chamber in 200 µL serum-free medium. Then the lower chambers were fueled with 500 µL full medium which containing 10% FBS. 48 hours later, the cells which did not move through the pores were carefully wiped out and cells on the undersurface of the chambers were fixed and stained.

### Establishment of stable cell lines

TTLL6/CDDP knockdown/overexpression stable cell lines were established by lentiviral transduction with indicated constructs. In brief, vectors pCMV-VSV-G plasmid (Addgene), pCMV-dR8.2 dvpr plasmid (Addgene) and pLenti-CMV-CHOP/pLKO.1-TTLL6-shRNA were co-transfected into HEK-293T packaging cells. After 48 hours incubation, the viral supernatant fraction was collected. Then, cells were infected with lentiviral particles for 24 hours following with over one week of puromycin selection. Plasmids were transected using Effectene Transfection Reagent (QIAGEN) according to the manufacturer's protocol.

### *In vitro* drug sensitivity assay (MTT assay)

Cells were seeded into a 96-well plate at 3×10^4^ cells/well in culture medium. Each sample had five replicates. Viable cells were counted by the MTT assay 48h after treatment with 2, 4, 6, 8 and 10 μg/mL of CDDP. Briefly, 50 µL of 0.2% MTT was added into each well following with 4 hours incubation at 37 °C with 5% CO_2_. Then the Formazan crystal was dissolve with 150 µL DMSO, and the absorbance at 570nm was measured by using a 96-well plate reader (Dynatech MR 5000, Dynex Technologies, Ashford, UK).

### Annexin-V/Propidium iodide staining

5 µL Annexin V-FITC (Pharmingen, San Diego, CA) and 10 µL of 20 µg/ml propidium iodide (PI) (Sigma) were added to indicated cells and resuspended in 100 µL binding buffer at a concentration of 1×10^6^/mL. After 15 min incubation at RT, 400 µL Annexin-binding buffer was added to each sample. The mean fluorescence intensity of Annexin-V-FITC/PI was determined by flow cytometry and the apoptosis index (AI) was calculated.

### RNA Interference (RNAi)

EC109/CDDP cells were grown overnight to approximately 70% confluence and transfected with human TTLL6 short interfering RNA (siRNA) siRNA-01, siRNA-02, siRNA-03 or non-targeting siRNA (FITC Conjugate)-A (sc-36869, Santa Cruz Biotechnology Inc., Santa Cruz, CA). Sequences of siRNAs are listed in **Table 1**. Transfection was performed with Lipofectamine RNAi MAX reagent (Invitrogen, Carlsbad, CA) according to the manufacturer's instructions.

### Total RNA isolation, cDNA synthesis and PCR

Total RNA was extracted by using QIAshredder and RNeasy Kit (QIAGEN) according to the manufacturer's protocol. 1.0 µg RNA was reverse-transcribed with the SuperScript™ III Reverse Transcriptase kit (Thermo Scientific) following the manufacturer's protocol. PCR was performed by using the Phusion reaction system (Thermo Scientific) and RT-PCR was performed by using rTaq system (Thermo Scientific) with the primers listed in **Table 2**.

### Immunoblotting analysis

Cells were lysed using cold NP40 buffer with proteinase inhibitor mixture (Sigma-Aldrich). Protein were resolved on SDS-PAGE and transferred to PVDF membrane (Invitrogen). After blocking with 5% milk in Tris-buffered saline plus Tween 20 (TBST) for 30 min, the membrane was incubated overnight at 4°C with primary antibody, followed by incubation with a corresponding horseradish peroxidase-conjugated secondary antibody (Santa Cruz Biotechnology). Membrane was developed in West Pico Super Signal chemiluminescent substrate (Pierce). Primary antibodies used were as follows: anti-beta actin, anti-N-Cadherin, anti-E-Cadherin, anti-TopoIIA and anti-Caspase 9 (Source: rabbit, diluted 1:1000, Santa Cruz Biotechnology, Inc.); anti-TTLL6 and anti-ERBB2 (Source: rabbit, diluted 1:1000, Cell Signaling Technology, Inc.).

### *In vivo* drug sensitivity assay

4-week old female BALB/c nude mice were purchased from the Beijing HFK Bio-Technology Co.Ltd. (Beijing, China) and kept in the animal center of Army Medical University (Chongqing, China). For *in vivo* chemosensitivity assays, indicated cells were subcutaneously inoculated into nude mice (6 mice per group, 1×10^6^ cells for each mouse). Tumor growth was examined every other day, and tumor volumes were calculated using the equation V = 0.52×A×B^2^ (mm^3^), A represented the largest diameter and B represented the perpendicular diameter. When the average tumor size reached 700 mm^3^, CDDP was administered by subcutaneous injection at a dose of 4mg/kg once every two days for three times in total. The tumor volumes were measured every two days for two weeks since the last CDDP treatment. Then all mice were sacrificed to harvest the tumor tissues to perform immunohistochemistry assay and hematoxylin & eosin (H&E) staining. All procedures involving animal were approved by the Committee on the Use and Care on Animals (Army Medical University, Chongqing, China). All animals received humane care according to the criteria outlined in the “Guide for the Care and Use of Laboratory Animals” prepared by the National Academy.

### Immunohistochemistry

Streptavidinbiotin peroxidase complex method was used for immunohistochemical staining on formalin fixed, paraffin-embedded tissue sections. Rabbit anti-human TopoIIA, ERBB2 and Caspase 9 antibody (1:200) were added for 1 hour at 37 °C and 4 °C overnight. Following several washes with PBS, the slides were incubated with the appropriate secondary antibody for 2 hours. Then signals were visualized using the 3,3'-diaminobenzidine substrate solution (DAKO) and light counterstaining with hematoxylin. Nonimmune rabbit immunoglobulin was used as a negative control for the primary antibodies.

### Statistical analysis

The statistical significance of differences for the mean values of groups was determined with Student's *t*-test. Data were expressed as means ± SD. All statistical analysis was performed using SPSS 11.0 software (SPSS Inc., Chicago, IL, USA). Differences with a* P* value of less than 0.05 were considered significant.

## Results

### Establishment of CDDP resistant EC109 cells

**Figure 1:**
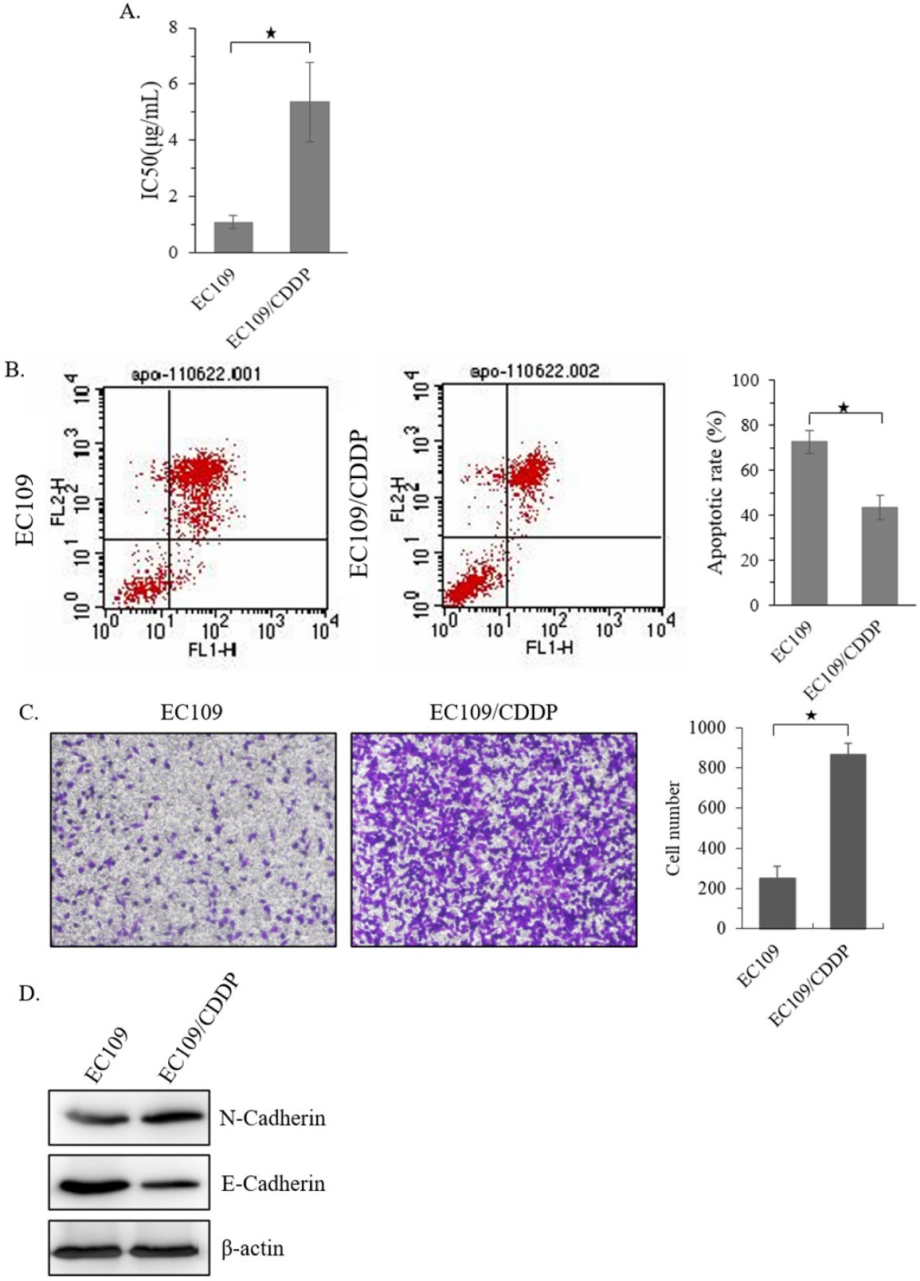
** Establishment of CDDP resistant EC109 cells.** (**A**) The IC_50_ of EC109 and EC109/CDDP cells. (**B**) Representative images of Annexin-V-FITC staining and the apoptosis index of EC109 and EC109/CDDP cells; *, *p* < 0.05. Data are means ± SD. from three independent experiments. (**C**) The invasion ability of EC109 and EC109/CDDP cells; *, *p* < 0.05 vs. EC109 cells. Data are means ± SD. from three independent experiments. (**D**) Indicated gene expression in EC109 and EC109/CDDP cells were detected by immunoblotting.

Firstly, a CDDP resistant sub-cell line of EC109 (EC109/CDDP) was established. MTT analysis showed that, compared with parental EC109 cells, the IC_50_ of EC109/CDDP cells increased form 1.15 ± 0.19 µg/mL to 5.19 ± 1.36 µg/mL **(Figure 1A)**. Next, the level of apoptosis in these cells was evaluated. After 48 hours treatment with 5 μg/mL of CDDP, the EC109/CDDP cells exhibited a significantly decreased apoptotic rate comparing with that in EC109 cells (41.2% ± 5.3% vs. 72.5% ± 4.9%) **(Figure 1B).** In addition, the invasion ability of EC109/CDDP cells was significantly increased **(Figure 1C)**, along with the reducing of E-cadherin expression and increasing of N-cadherin expression** (Figure 1D)**. Our data suggested a CDDP resistant EC109-sub cells line was successfully generated.

### Elevated TTLL6 expression sensitizes EC109/CDDP cells to drug treatment in hypoxia/acidosis microenvironment

**Figure 2:**
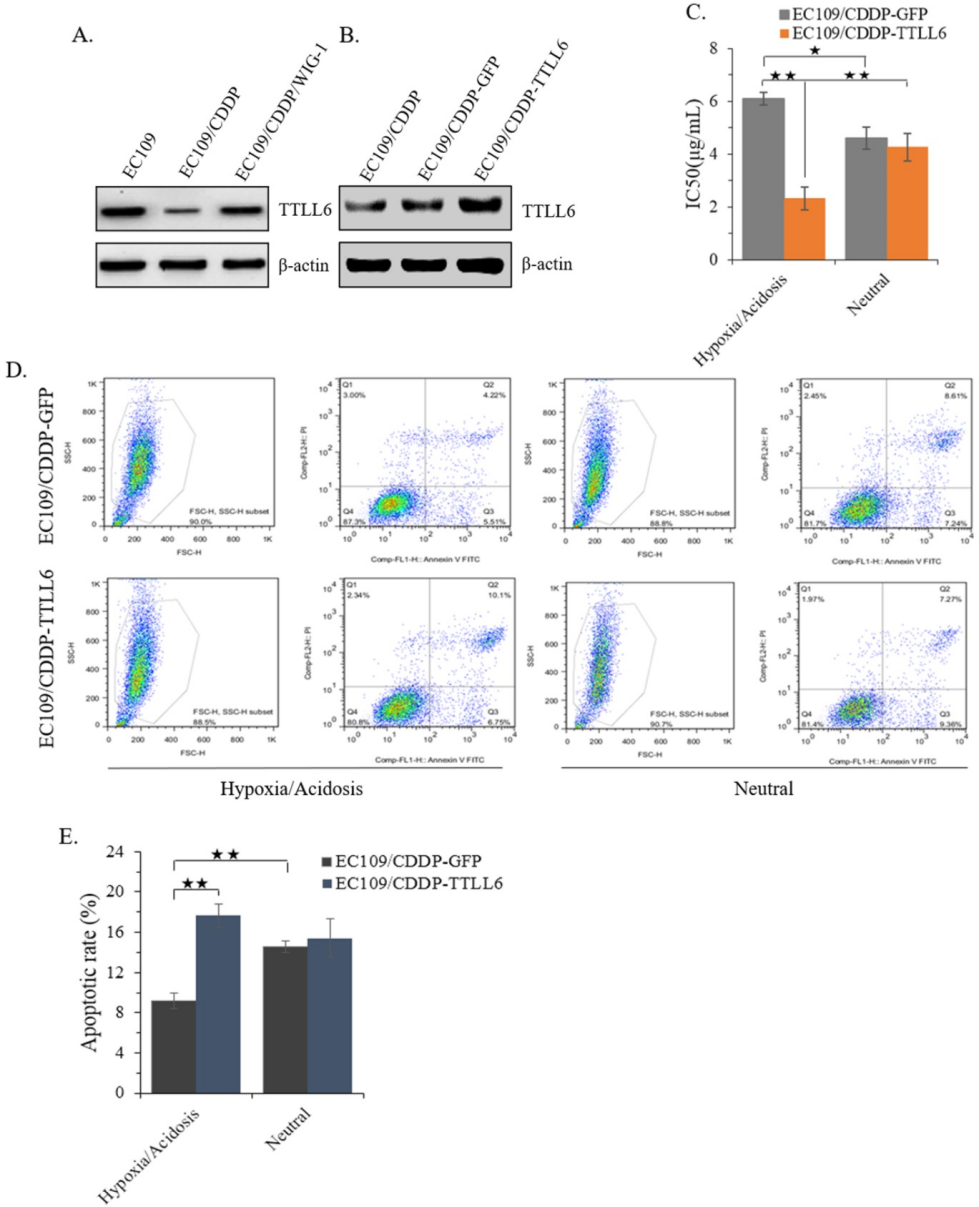
** Effect of overexpression TTLL6 on CDDP resistance and apoptosis of EC109/CDDP cells in Hypoxia/Acidosis Microenvironment.** (**A**) WIG-1 reversed EC109/CDDP cells increased TTLL6 expression. (**B**) The elevated TTLL6 expression in EC109/CDDP-TTLL6 cells comparing with parental control and EC109/CDDP-GFP control cells. (**C**) The IC_50_ value of EC109/CDDP-TTLL6 and EC109/CDDP-GFP control cells cultured in neutral or hypoxia/acidosis microenvironment; *, *p* < 0.05, **, *p* < 0.01. Data are means ± SD. from three independent experiments. Representative images showed the Annexin-V/Propidium Iodide staining (**D**) and the apoptosis index (**E**) of EC109/CDDP-TTLL6 and EC109/CDDP-GFP control cells cultured in neutral or hypoxia/acidosis microenvironment; **, *p* < 0.01. Data are means ± SD. from three independent experiments.

In our previous study, we reported that stably enhanced WIG-1 expression could reverse the drug resistance of EC109/CDDP cells 16. Here we found the TTLL6 expression was decreased in EC109/CDDP cells compared with parental EC109 cells; while in the EC109/CDDP/WIG-1 cells, the TTLL6 expression was elevated compared with EC109/CDDP cells **(Figure 2A)**. Our data indicated that TTLL6 may play important roles in reversing the drug resistance. To explore the role of TTLL6 in the CDDP resistance of EC cells, TTLL6 stable overexpressed EC109/CDDP cells were established. Western blot result showed the EC109/CDDP-TTLL6 cells increased the expression of TTLL6 comparing with EC109/CDDP and EC109/CDDP-GFP control cells **(Figure 2B)**. Considering the human esophageal cancer cells growth and the occurrence of drug resistance generally occurred in hypoxia/acidosis microenvironment. Therefore, in order to more veritably reveal the role of TTLL6 in drug resistant EC cells, we performed our following study in Hypoxia/Acidosis (O_2_ 1%; pH 4.0) and neutral (O_2_ 21%; pH 6.8) microenvironment. MTT analysis suggested that overexpression of TTLL6 significantly lowered the IC_50_ of EC109/CDDP-TTLL6 cells in comparison with EC109/CDDP-GFP cells (2.41 ± 0.38 µg/mL vs. 6.09 ± 0.19 µg/mL) in hypoxia/acidosis but not in neutral microenvironment **(Figure 2C)**. Interestingly, the EC109/CDDP-GFP cells cultured in hypoxia/acidosis microenvironment had a higher IC_50_ value than in neutral microenvironment (6.09 ± 0.19 µg/mL vs. 4.62 ± 0.37 µg/mL); while the IC_50_ value of EC109/CDDP-TTLL6 cells in hypoxia/acidosis microenvironment is much lower than in neutral microenvironment (2.41 ± 0.38 µg/mL vs. 4.18 ± 0.51 µg/mL) **(Figure 2C)**. Next the effect of TTLL6 on CDDP-induced apoptosis was measured. After 24 hours treatment of set cells with 2.5 μg/mL of CDDP the flow cytometry analysis was performed. Our data showed that, as compared with GFP overexpression cells, TTLL6 overexpression cells had a higher CDDP-induced apoptosis rate in hypoxia/acidosis (17.7% ± 1.14% vs.9.82% ± 0.77%). While in neutral microenvironment the apoptosis rates had no significant differences **(Figure 2D and E)**. Moreover, for EC109/CDDP-GFP cells, hypoxia/acidosis microenvironment could reduce CDDP-induced apoptosis (9.82% ± 0.77% vs. 15.4% ± 0.61%); while for EC109/CDDP-TTLL6 cells, the two microenvironments made no significant difference of CDDP-induced apoptosis rate **(Figure 2D and E)**. These data indicated that increasing of TTLL6 expression could enhance the therapeutic effect of drugs for drug-resistant EC cells in hypoxia/acidosis microenvironment.

### TTLL6 deficiency desensitizes EC109/CDDP cells to drug treatment in hypoxia/acidosis microenvironment

**Figure 3:**
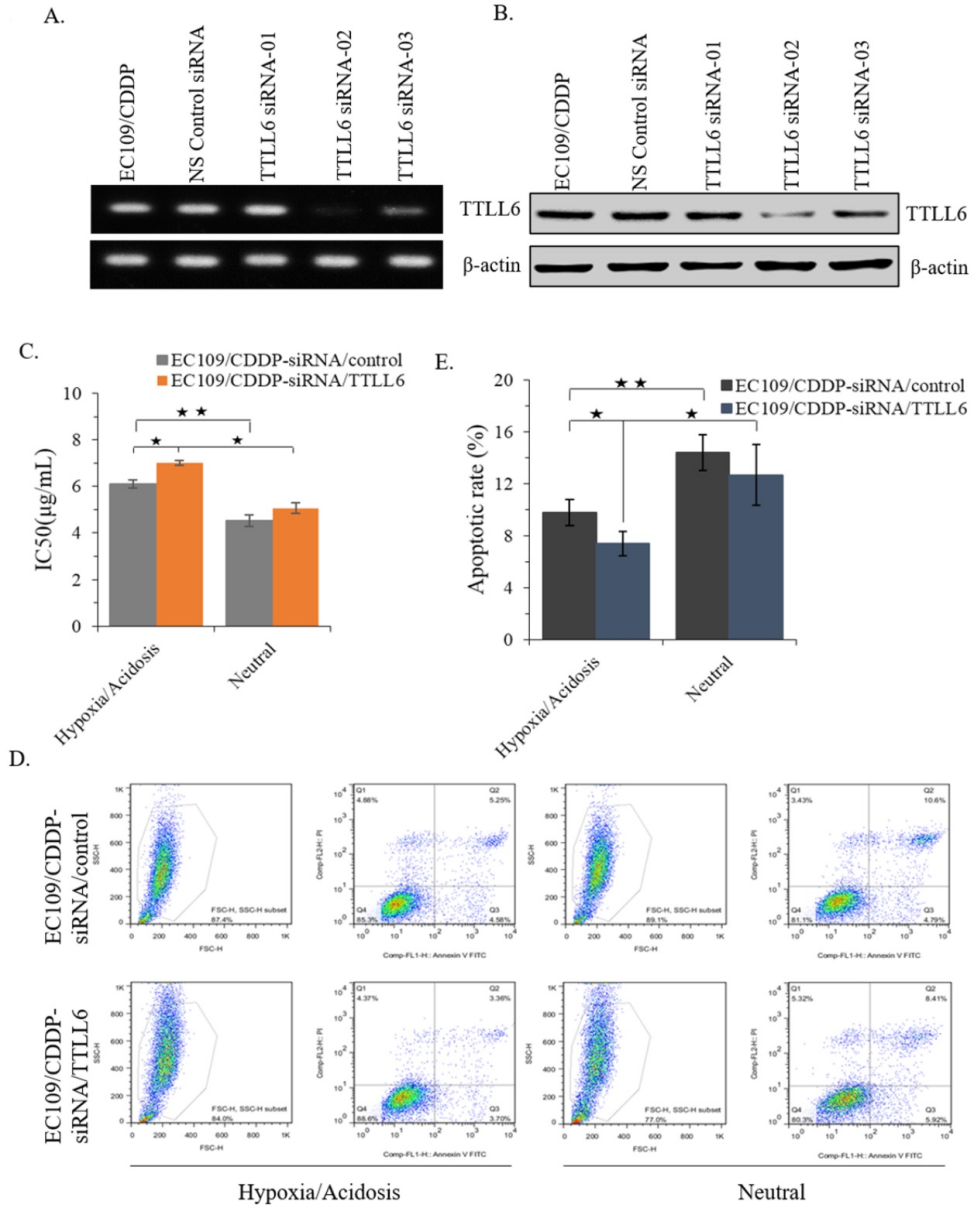
** Effect of silencing TTLL6 expression on CDDP resistance and apoptosis of EC109/CDDP cells in hypoxia/acidosis microenvironment.** Semi-QPCR (**A**) and western blot (**B**) analysis the knockdown efficiency of three selected siRNAs of TTLL6 gene. (**C**) The IC_50_ value of EC109/CDDP-siRNA/TTLL6 and EC109/CDDP-siRNA/control cells cultured in neutral or hypoxia/acidosis microenvironment; *, *p* < 0.05, **, *p* < 0.01. Data are means ± SD. from three independent experiments. Representative images showed the Annexin-V/Propidium Iodide staining (**D**) and the apoptosis index (**E**) of EC109/CDDP-siRNA/TTLL6 and EC109/CDDP-siRNA/control cells cultured in neutral or hypoxia/acidosis microenvironment; *, *p* < 0.05, **, *p* < 0.01. Data are means ± SD. from three independent experiments.

To further investigate the role of TTLL6 in regulation of CDDP resistance of EC cells, we selected one TTLL6 siRNA sequence (TTLL6 siRNA-02) which achieved a potent loss of TTLL6 expression both at mRNA and protein level **(Figure 3A and B)**. Next, IC_50_ analysis revealed that silencing TTLL6 expression significantly increased the IC_50_ of EC109/CDDP-siRNA/TTLL6 cells in comparison with EC109/CDDP-siRNA/control cells (7.01 ± 0.11 µg/mL vs. 6.02 ± 0.17 µg/mL) in hypoxia/acidosis but not neutral microenvironment **(Figure 3C)**. In addition, both IC_50_ of EC109/CDDP-siRNA/control and EC109/CDDP-siRNA/TTLL6 cells increased in hypoxia/acidosis microenvironment comparing with in neutral microenvironment (6.02 ± 0.17 µg/mL vs. 4.51 ± 0.24 µg/mL and 7.01 ± 0.11 µg/mL vs. 5.06 ± 0.23 µg/mL) **(Figure 3C)**. Furtherly, we investigated the effect of TTLL6 deficiency on CDDP-induced apoptosis in EC109/CDDP cells. After 24 hours treatment of set cells with 4.5 μg/mL of CDDP the flow cytometry analysis was performed. Our results showed that, comparing with siRNA control, TTLL6 silencing decreased the CDDP-induced apoptosis in hypoxia/acidosis but not neutral microenvironment (9.77% ± 0.98% vs. 7.41% ± 0.94%) **(Figure 3D and E)**. Moreover, both apoptosis rate of EC109/CDDP-siRNA/control and EC109/CDDP-siRNA/TTLL6 cells reduced in hypoxia/acidosis microenvironment comparing with in neutral microenvironment (9.77% ± 0.98% vs. 14.4% ± 1.39% and 7.41 ± 0.94 µg/mL vs. 12.66 ± 2.36 µg/mL) **(Figure 3D and E)**. These data suggested that reduced expression of TTLL6 may play a critical role in the development of drug resistance for EC cells in hypoxia/acidosis microenvironment.

### Effect of TTLL6 on expression of drug resistance related molecules

**Figure 4:**
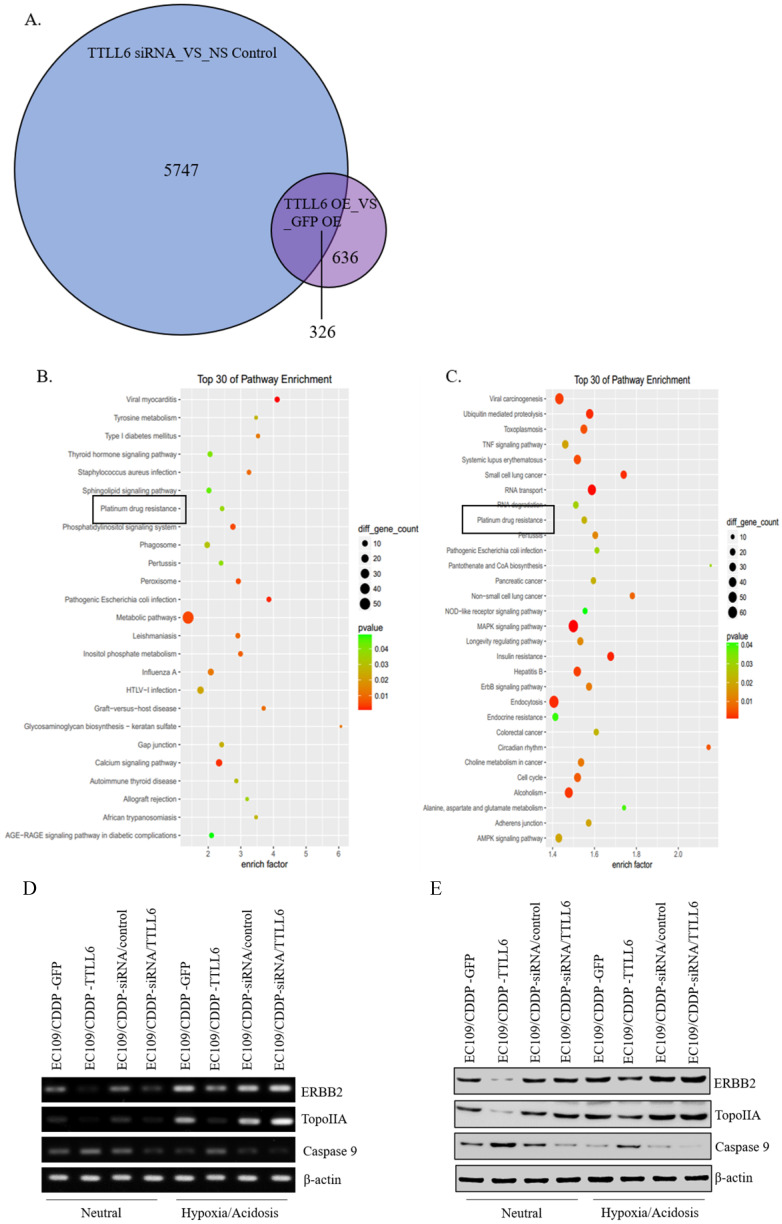
** TTLL6 regulated the drug resistance related molecules.** (**A**) The number of genes which were affected by TTLL6 downregulation or overexpression. (**B**) The enriched molecular pathways presented by genes significantly affected by TTLL6 downregulation in EC109/CDDP cells. (**C**) The enriched molecular pathways presented by genes significantly affected by TTLL6 overexpression in EC109/CDDP cells. Semi-QPCR (**D**) and western blot (**E**) analysis the expression of indicated genes while the TTLL6 expression of EC109/CDDP cells were alternated and cells were cultured in neutral or hypoxia/acidosis microenvironment.

To elucidate the mechanisms by which TTLL6 promotes the sensitivity of the esophageal carcinoma cells to drug treatment, we examined global gene expression profiles in EC109/CDDP cells which were infected with Lv-TTLL6 or Lv-GFP as well as transfected with siRNA against TTLL6 or control siRNA by cDNA microarray. We found that there were 5747 genes with 2 folds or higher change in their expression when TTLL6 was knocked down and 636 genes changed when TTLL6 was overexpressed; Among them, 326 genes were intersectional **(Figure 4A)**. SBC Analysis System analysis showed that both these genes enriched in the pathway of platinum drug resistance which involves gene TopoIIA, Caspase 9 and ERBB2 **(Figure 4B and C).** Then, the expression of TopoIIA, Caspase 9 and ERBB2 were evaluated in drug resistant EC cells by semi-quantitative RT-PCR and western blot analysis to further elucidate the mechanisms involved in TTLL6-dependent CDDP resistance. Following the upregulation of TTLL6 in EC109/CDDP cells cultured in neutral or hypoxia/acidosis microenvironment, the expression of ERBB2 and TopoIIA were significantly reduced, while the opposite result was observed for Caspase 9; When the expression of TTLL6 was silenced in EC109/CDDP cells, the expression of TopoIIA was enhanced, while the expression of Caspase 9 was depressed. However, we did not observe significant alteration of ERBB2 expression when TTLL6 expression was inhibited. Moreover, compared with in neutral microenvironment, EC109/CDDP cells in hypoxia/acidosis microenvironment increased the expression of ERBB2 and TopoIIA, but reduced the Caspase 9 expression **(Figure 4D and E)**. In addition, the expression of GST and p170 were not influenced by either alteration of TTLL6 expression or alteration of the culture microenvironment (data not show). These data demonstrated that TTLL6 may play an important role in developing CDDP resistance by regulating the expression of TopoIIA, Caspase 9 and ERBB2 in EC cells.

### Effect of TTLL6 on the CDDP sensitivity of xenografts in animal model

**Figure 5:**
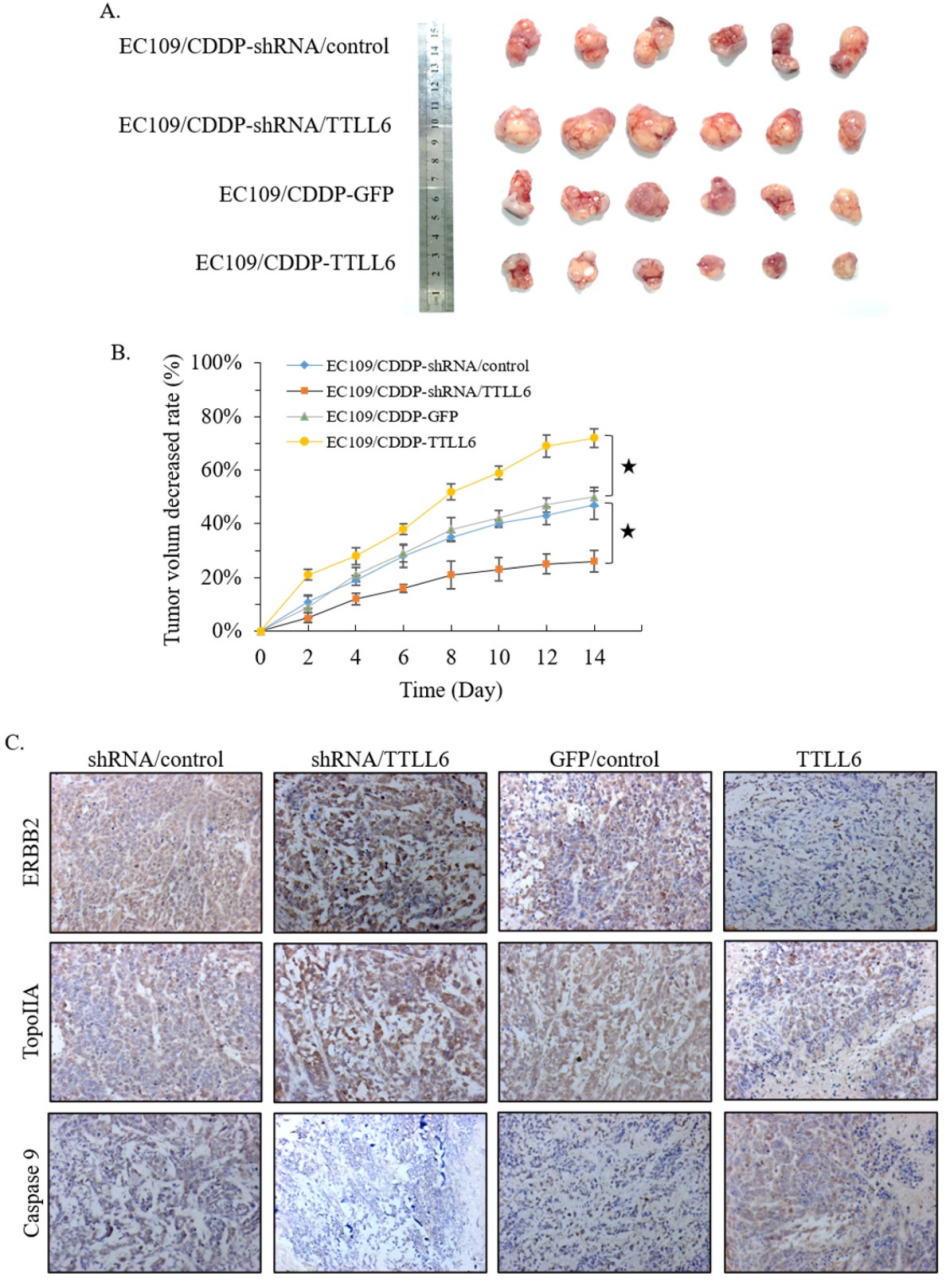
** Effect of TTLL6 on the CDDP sensitivity of xenografts *in vivo*.** (**A**) Established groups of EC109/CDDP xenografts were surgically removed 20 days after initial treatment. (**B**) The average reduced rates of tumor volume in indicated groups; *, *p*<0.01 versus EC109/CDDP-shRNA/control or EC109/CDDP-GFP control group. (**C**) Representative images showed the expression of indicated genes analyzed by immunohistochemical on tumor xenografts (magnification × 200).

To further investigate the role of TTLL6 in the resistance of chemotherapy, we first selected TTLL6 stable knockdown cells (EC109/CDDP-shRNA/TTLL6) by lentiviral infection, and EC109/CDDP-shRNA/control cells were served as control. Together with EC109/CDDP-GFP and EC109/CDDP-TTLL6 cells, we established four groups of EC109/CDDP xenografts. When the average tumor size reached 700 mm^3^, CDDP was administered by subcutaneous injection at a dose of 4 mg/kg once every two days for three times in total. 2 weeks after treatment, the tumor size of EC109/CDDP-shRNA/TTLL6 group was larger than that in EC109/CDDP-shRNA/control group; while the tumor size of EC109/CDDP-TTLL6 group was smaller than that in the EC109/CDDP-GFP control group** (Figure 5A)**. The average reduced rate of tumor volume was significantly lower in TTLL6 overexpressed group than that in GFP overexpressed control group, and it was significantly higher in TTLL6 silenced group than that in shRNA control group **(Figure 5B)**. These data suggested that expression of TTLL6 can effectively increase the CDDP sensitivity or even reverse the CDDP resistance of EC109/CDDP cells *in vivo*. To further corroborate these results, the expression of TopoIIA, Caspase 9 and ERBB2 were evaluated by immunohistochemistry. The silencing of TTLL6 significantly upregulated ERBB2 as well as TopoIIA expression and inhibited Caspase 9 expression, while overexpression of TTLL6 reduced the expression of ERBB2 and TopoIIA but enhanced the expression of Caspase 9 **(Figure 5C)**. These data thus suggested that TTLL6 played an important role in CDDP sensitivity of EC/CDDP cells *in vivo*.

## Discussion

The coding sequence of tubulin tyrosine ligase-like family member 6 (TTLL6) locates in the chromosomal locus17q21.32. It has been reported the 3' UTR of TTLL6 lies within the distal breakpoint region of *De novo* t(12;17) (p13.3; q21.3) translocation in a patient with developmental delay and skeletal malformations 17. However, whether alteration of TTLL6 expression directly caused these syndromes is still unknown. The most studied and well-known functions of TTLL6 lie in stabilization and motility of ependymal cilia 18, 19 and microtubules (MTs) stability 20. Studies have confirmed that the change of microtubules stability and interactions between microtubules and cytoskeletal proteins (γ-actin or profilin) were involved in the development of MDR to tumors 21, 22. In our previous study we occasionally found that TTLL6 expression was significantly increased in reversed EC109/CDDP cells. Therefore, TTLL6 may paly critical roles in the development of MDR and may be a potential candidate for drug resistant esophageal carcinoma therapy.

To further investigate the effect of TTLL6 on the drug resistance of EC cells, we first stably overexpressed TTLL6 in EC/CDDP cells. Elevated TTLL6 expression enhanced the cellular sensitivity to CDDP and increased the CDDP induced apoptosis only in hypoxia/acidosis microenvironment cultured EC/CDDP cells (Figure 2). Then using siRNA to silence the expression of TTLL6, we observed the opposite effects. Knockdown of TTLL6 expression desensitized EC109/CDDP cells to CDDP and decreased the CDDP induced apoptosis only in hypoxia/acidosis microenvironment cultured EC/CDDP cells. Our data indicated that TTLL6 palys critical roles in CDDP resistance of EC/CDDP cells in hypoxia/acidosis microenvironment. Next the molecular mechanism analysis revealed that TTLL6 regulated the expression of ERBB2, TopoIIA and Caspase 9. ERBB2 is well known as a key molecular in the multi-drug resistance of breast cancer 23, 24. As early as 2003, Masayuki Akamatsu et al. have concluded that ERBB2 could be useful for the prediction of chemoradioresistance in esophageal squamous cell carcinoma 25. However, there are barely any further reports about its functions in drug resistance of esophageal cancer. It has been reported that TOPOIIA may predict the treatment outcome in patients with lung cancer and gastric cancer 26, 27. Those genes are involving in the development of drug resistance and apoptosis 28-31 and those results were in accordance with our previous study in WIG-1 reversed EC109/CDDP cells 16. However, the exact regulation mechanism needs to be further revealed. Interestingly, the expression level of ERBB2 and TopoIIA in hypoxia/acidosis cultured EC/CDDP cells were much higher than that in neutral microenvironment cultured EC/CDDP cells; and the expression level of Caspase 9 exhibited the opposite result. Those results may explain why there were no significantly effect on the IC_50_ and apoptotic rate of EC/CDDP cells cultured in neutral microenvironment when the TTLL6 expression were alternated. The animal experiment results also confirmed that TTLL6 overexpression increased the treatment effect of CDDP. Those data directly provided further evidence that TTLL6 could be used to development therapeutic strategy to reverse drug resistance of tumors.

In summary, our results demonstrated that the TTLL6-dependent reversal of CDDP resistance in EC109/CDDP cells partly due to alternating the expression of certain drug resistance-related proteins, including TopoIIA, ERBB2 and Caspase 9. Although the biological functions of TTLL6 in esophageal cancer require further study, our data provided first inspection of TTLL6 deficiency induced CDPP resistance and provided an intervention strategy for drug resistance reversal of esophageal cancer chemotherapy.

## Figures and Tables

**Table 1:** The oligonucleotide sequence of siRNA and shRNA for silencing TTLL6 expression

TTLL6	Sequence (5' to 3')
siRNA-01	CGAGAUUGGTGAUCAAUCUdTdT
siRNA-02	CGGACUCATGAUUUCCAGGAdTdT
siRNA-03	CGCAUACAGCCAUUGCCUGAdTdT
shRNA	CCGGTCGCCACATTTGATATCCGAGCGGGATCCAACTCGGATATCAAATGTGGCGTTTTTG
sh Control	CCGGTACCTCACACTGTAGGTCGTACGGGATCCAATACGACCTACAGTGTGAGGTTTTTTG

**Table 2:** The oligonucleotide primers used in the PCR reaction

Gene	Direction	Primer sequence (5' to 3')
TTLL6/cDNA	Forward	CGCGGATCC ATGAGAATTGGCACCCCAACA BamH I
	Reverse	ACGCGTCGACCTAGCTCCTCTCACATCCTTTTT Sal I
TTLL6	Forward	TCATCATCAAGACCCTCATCTCGGC
	Reverse	TCTTTAACTGCACGGCCCGGAAAC
GST	Forward	CCGCCCTACACCGTGGTCTAT
	Reverse	GCTGCCTCCTGCTGGTCCTT
ERBB2	Forward	ACGCCGAATATGCCATCTCAC
	Reverse	AGCCGCCCATGGATGTAGTCT
P170	Forward	GCCATAGCTCGTGCCCTTGTT
	Reverse	GCGTGCCATGCTCCTTGACTC
MRP	Forward	CACGACCTCCGCTTCAAGATC
	Reverse	CGCCTTCTGCACATTCATGGT
Topo IIA	Forward	GGAGAGCAGCAACAAAAACAAAAT
	Reverse	AGGAGGGCTTGAAGACAGTGGTA
Caspase 9	Forward	GCCGCCGTGGACACAGAC
	Reverse	GGGCCTTGAGCACCAGTTTG
β-actin	Forward	GCCGACAGGATGCAGAAGGAGAT
	Reverse	GGGGCCGGACTCGTCATACT

## References

[1] Pan R, Zhu M, Yu C, Lv J, Guo Y, Bian Z, Yang L, Chen Y, Hu Z, Chen Z, et al.Cancer incidence, mortality (2017). A cohort study in China, 2008-2013. Int J Cancer.

[2] Feng RM, Zong YN, Cao SM, Xu RH (2019). Current cancer situation in China: good or bad news from the 2018 Global Cancer Statistics?. Cancer Commun.

[3] Yuan LG, Mao YS (2019). Current status of prognostic evaluation of esophageal cancer patients by circumferential resection margin. Zhonghua Zhong Liu Za Zhi.

[4] Kang X, Chen K, Li Y, Li J, D'Amico TA, Chen X (2015). Personalized targeted therapy for esophageal squamous cell carcinoma. World journal of gastroenterology.

[5] Hede K (2004). Environmental protection: studies highlight importance of tumor microenvironment. Journal of the National Cancer Institute.

[6] Nordsmark M, Loncaster J, Aquino-Parsons C, Chou SC, Gebski V, West C, Lindegaard JC, Havsteen H, Davidson SE, Hunter R (2006). The prognostic value of pimonidazole and tumour pO2 in human cervix carcinomas after radiation therapy: a prospective international multi-center study. Radiotherapy and oncology: journal of the European Society for Therapeutic Radiology and Oncology.

[7] Hartmann P, Mirtolouei R, Untersberger S, Ziegler W, Hermann Z, Richtig E, Hofmann-Wellenhof R, Grinschgl S, Kerl H, Smolle J (2006). Non-invasive imaging of tissue PO2 in malignant melanoma of the skin. Melanoma research.

[8] Webb BA, Chimenti M, Jacobson MP, Barber DL Dysregulated pH: a perfect storm for cancer progression: Nat Rev Cancer. 2011; 11(9):671-7. doi: 10.1038/nrc3110.

[9] Idriss HT (2004). Three steps to cancer: how phosphorylation of tubulin, tubulin tyrosine ligase and P-glycoprotein may generate and sustain cancer. Cancer chemotherapy and pharmacology.

[10] Barisic M, Silva e Sousa R, Tripathy SK, Magiera MM, Zaytsev AV, Pereira AL, Janke C, Grishchuk EL, Maiato H (2015). Mitosis. Microtubule detyrosination guides chromosomes during mitosis. Science.

[11] Tanaka N, Horie N, Kusajima K, Kitano I, Komatsu S (1976). Surgical management of the tricuspid valve-follow-up studies of valve replacement and annuloplasty. Zasshi Journal Nihon Kyobu Geka Gakkai.

[12] Ota T, Suzuki Y, Nishikawa T, Otsuki T, Sugiyama T, Irie R, Wakamatsu A, Hayashi K, Sato H, Nagai K (2004). Complete sequencing and characterization of 21,243 full-length human cDNAs. Nature genetics.

[13] McClusky LM (2005). Stage and season effects on cell cycle and apoptotic activities of germ cells and Sertoli cells during spermatogenesis in the spiny dogfish (Squalus acanthias). Reproduction.

[14] Sawhney P, Giammona CJ, Meistrich ML, Richburg JH (2005). Cisplatin-induced long-term failure of spermatogenesis in adult C57/Bl/6J mice. Journal of andrology.

[15] Guo W, Zou YB, Jiang YG, Wang RW, Zhao YP, Ma Z (2011). Zinc induces cell cycle arrest and apoptosis by upregulation of WIG-1 in esophageal squamous cancer cell line EC109. Tumour biology: the journal of the International Society for Oncodevelopmental Biology and Medicine.

[16] Qiu Y, Zou YB, Li K, Jiang YG, Yang K, Zhao YP, Guo W (2012). Effect of altered WIG-1 expression on DDP sensitivity in a DDP-resistant esophageal squamous cancer cell line. Current cancer drug targets.

[17] Yue Y, Farcas R, Thiel G, Bommer C, Grossmann B, Galetzka D, Kelbova C, Kupferling P, Daser A, Zechner U (2007). *De novo* t(12;17)(p13.3;q21.3) translocation with a breakpoint near the 5' end of the HOXB gene cluster in a patient with developmental delay and skeletal malformations. European journal of human genetics: EJHG.

[18] Suryavanshi S, Edde B, Fox LA, Guerrero S, Hard R, Hennessey T, Kabi A, Malison D, Pennock D, Sale WS (2010). Tubulin glutamylation regulates ciliary motility by altering inner dynein arm activity. Current biology.

[19] Bosch Grau M, Gonzalez Curto G, Rocha C, Magiera MM, Marques Sousa P, Giordano T, Spassky N, Janke C (2013). Tubulin glycylases and glutamylases have distinct functions in stabilization and motility of ependymal cilia. The Journal of cell biology.

[20] Zempel H, Luedtke J, Kumar Y, Biernat J, Dawson H, Mandelkow E, Mandelkow EM (2013). Amyloid-beta oligomers induce synaptic damage via Tau-dependent microtubule severing by TTLL6 and spastin. The EMBO journal.

[21] Kavallaris M (2010). Microtubules and resistance to tubulin-binding agents. Nature reviews Cancer.

[22] Kavallaris M, Annereau JP, Barret JM (2008). Potential mechanisms of resistance to microtubule inhibitors. Seminars in oncology.

[23] Yue LU, Xiang JY, Sun P, Yao YS, Sun ZN, Liu XP, Wang HB, Shen Z, Yao RY (2016). Relationship Between HSP70 and ERBB2 Expression in Breast Cancer Cell Lines Regarding Drug Resistance. Anticancer Res.

[24] Ruprecht B, Zaal EA, Zecha J, Wu W, Berkers CR, Kuster B, Lemeer S (2017). Lapatinib Resistance in Breast Cancer Cells Is Accompanied by Phosphorylation-Mediated Reprogramming of Glycolysis. Cancer Res.

[25] Akamatsu M, Matsumoto T, Oka K, Yamasaki S, Sonoue H, Kajiyama Y, Tsurumaru M, Sasai K (2003). c-erbB-2 oncoprotein expression related to chemoradioresistance in esophageal squamous cell carcinoma. Int J Radiat Oncol Biol Phys.

[26] Karachaliou N, Papadaki C, Lagoudaki E, Trypaki M, Sfakianaki M, Koutsopoulos A, Mavroudis D, Stathopoulos E, Georgoulias V, Souglakos J (2013). Predictive value of BRCA1, ERCC1, ATP7B, PKM2, TOPOI, TOPΟ-IIA, TOPOIIB and C-MYC genes in patients with small cell lung cancer (SCLC) who received first line therapy with cisplatin and etoposide. PLoS One.

[27] Zha Y, Cun Y, Zhang Q, Li Y, Tan J (2012). Prognostic value of expression of Kit67, p53, TopoIIa and GSTP1 for curatively resected advanced gastric cancer patients receiving adjuvant paclitaxel plus capecitabine chemotherapy. Hepatogastroenterology.

[28] Okumura H, Uchikado Y, Setoyama T, Matsumoto M, Owaki T, Ishigami S, Natsugoe S (2014). Biomarkers for predicting the response of esophageal squamous cell carcinoma to neoadjuvant chemoradiation therapy. Surgery today.

[29] Arcy ND, Gabrielli B (2017). Topoisomerase II Inhibitors and Poisons, and the Influence of Cell Cycle Checkpoints. Current med chem.

[30] Paul I, Jones JM (2014). Apoptosis block as a barrier to effective therapy in non-small cell lung cancer. World journal of clinical oncology.

[31] Lu J, Zhou K, Yin X, Xu H, Ma B (2019). Molecular insight into the T798M gatekeeper mutation-caused acquired resistance to tyrosine kinase inhibitors in ErbB2-positive breast cancer. Computational Biology and Chemistry.

